# Supplementary figures and images for: Evoked potentials as biomarkers of hereditary spastic paraplegias: A case-control study
Source: PLoS One. 2021 Nov 30;16(11):e0259397. doi: 10.1371/journal.pone.0259397 (PMC8631666; doi:10.1371/journal.pone.0259397)

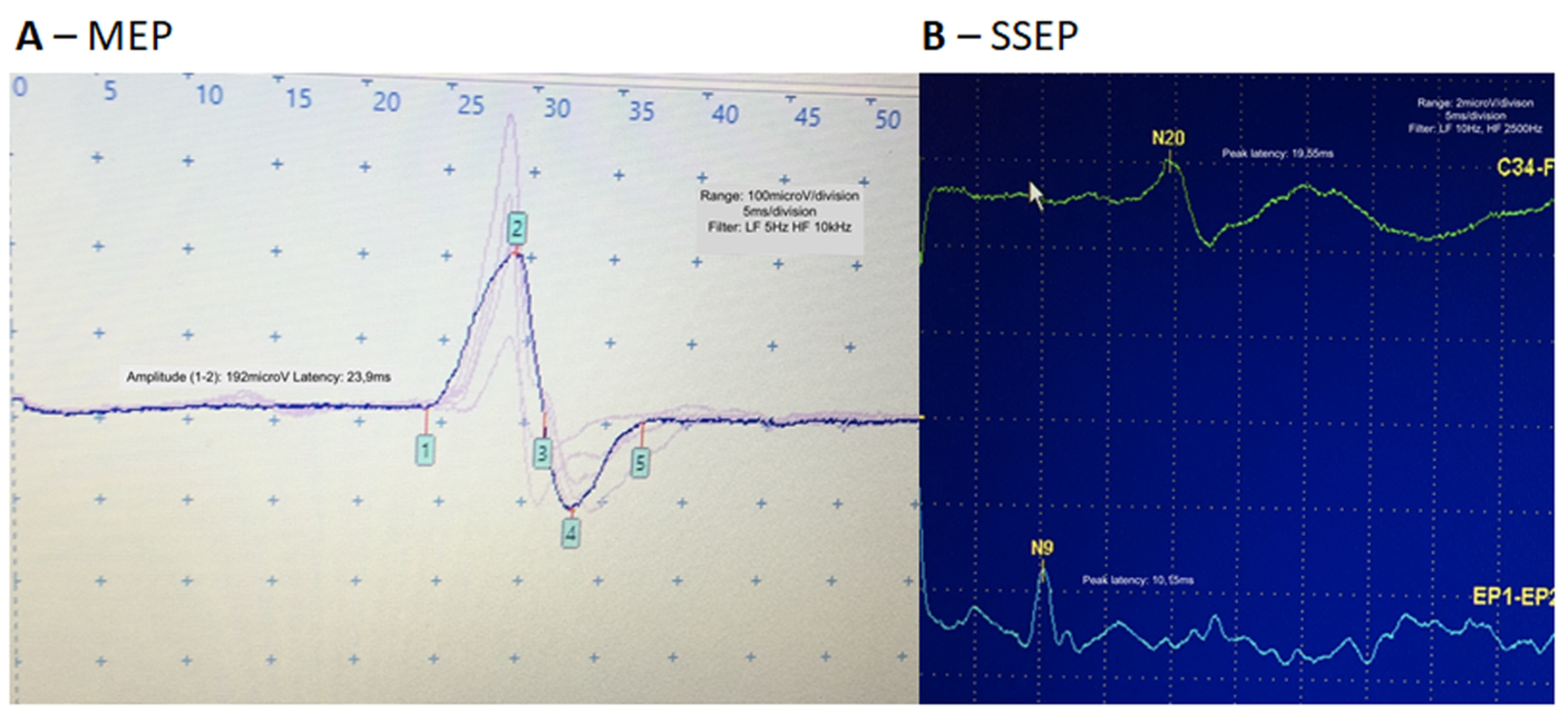

Supplement: S1 Fig — A) Example of a motor evoked potential (MEP); B) Example of a Somatosensory Evoked Potential (SSEP). (TIF) [file pone.0259397.s002.tif]
