# Supplementary material for: Evoked potentials as biomarkers of hereditary spastic paraplegias: A case-control study
Source: PLoS One. 2021 Nov 30;16(11):e0259397. doi: 10.1371/journal.pone.0259397 (PMC8631666; doi:10.1371/journal.pone.0259397)
Supplement: S1 Table — CMCT: Central Motor Conduction Time; HSP: Hereditary spastic paraplegias; LL: lower limbs; MEP: motor evoked potential; ms: milliseconds; mV: millivolt; SSEP: Somatosensory Evoked Potential; UL: upper limbs; μV: microvolt. (DOCX) [file pone.0259397.s003.docx]

**Supplemental Table 1 – Correlations of evoked potentials with clinical findings in the overall HSP group**

|  | **Age at onset (years)** | **Disease duration (years)** | **SPRS** | **SPRS motor** |
| --- | --- | --- | --- | --- |
| **MEP Amplitude UL (µV)** | Rho=0.07  p=0.78 | Rho=0.1  p=0.71 | Rho=0.08  p=0.75 | Rho=0.38  p=0.89 |
| **MEP Amplitide LL (µV)** | Rho=0.095  p=0.78 | Rh0=0.04  p=0.9 | Rho=0.17  p=0.6 | Rho=0.17  p=0.96 |
| **CMCT UL (ms)** | Rho=0.01  p=0.96 | Rho=0.08  p=0.73 | Rho=0.41  p=0.08 | Rho=0.38  p=0.13 |
| **CMCT LL (ms)** | Rho=0.51  p=0.07 | Rho=0.36  p=0.22 | Rho=0.49  p=0.08 | Rho=0.44  p=0.14 |
| **SSEP UL (ms)** | **Rho=0.61**  **p=0.01** | Rho=0.36  p=0.16 | Rho=0.45  p=0.08 | Rho=0.47  p=0.06 |
| **SSEP LL (ms)** | Rho=0.31  p=0.25 | **Rho=0.83**  **p=0.000** | **Rho=0.48**  **p=0.06** | **Rho= 0.50**  **p=0.056** |

**CMCT**: Central Motor Conduction Time; **HSP**: Hereditary spastic paraplegias; **LL**: lower limbs; **MEP:** motor evoked potential; **ms**: milliseconds; **mV**: millivolt; **SSEP**: Somatosensory Evoked Potential; **UL**: upper limbs; **µV:** microvolt.
