# Supplementary material for: Evoked potentials as biomarkers of hereditary spastic paraplegias: A case-control study
Source: PLoS One. 2021 Nov 30;16(11):e0259397. doi: 10.1371/journal.pone.0259397 (PMC8631666; doi:10.1371/journal.pone.0259397)
Supplement: S2 Table — CMCT: Central Motor Conduction Time; HSP: Hereditary spastic paraplegias; MEP: motor evoked potential; ms: milliseconds; mV: millivolt; SSEP: Somatosensory Evoked Potential; UL: upper limbs; μV: microvolt. (DOCX) [file pone.0259397.s004.docx]

**Supplemental Table 2 – Correlations of evoked potentials with clinical findings in the SPG4 subgroup**

|  | **Age at onset (years)** | **Disease duration (years)** | **SPRS** | **SPRS motor** |
| --- | --- | --- | --- | --- |
| **MEP Amplitude UL (µV)** | Rho=0.55  p=0.88 | Rho=0.22  p=0.53 | Rho=0.05  p=0.88 | Rho=0.02  p=0.94 |
| **MEP Amplitude LL (µV)** | Rh=0.3  p=0.55 | Rh0=0.04  p=0.84 | Rho=0.3  p=0.55 | Rho=0.6  p=0.87 |
| **CMCT UL (ms)** | Rho=0.16  p=0.60 | Rho=0.12  p=0.70 | Rho=0.35  p=0.25 | Rho=0.29  p=0.35 |
| **CMCT LL (ms)** | Rho=0.67  p=0.06 | Rho=0.37  p=0.35 | Rho=0.60  p=0.10 | Rho=0.52  p=0.18 |
| **SSEP UL (ms)** | **Rho=0.72**  **p=0.008** | Rho=0.43  p=0.16 | Rho=0.41  p=0.18 | Rho=0.41  p=0.17 |
| **SSEP LL (ms)** | Rho=0.31  p=0.34 | **Rho=0.81**  **p=0.002** | Rho=0.56  p=0.07 | Rho= 0.56  p=0.07 |

**CMCT**: Central Motor Conduction Time; **HSP**: Hereditary spastic paraplegias; **LL**: lower limbs; **MEP:** motor evoked potential; **ms**: milliseconds; **mV**: millivolt; **SSEP**: Somatosensory Evoked Potential; **UL**: upper limbs; **µV**: microvolt.
